# Evaluating the QUIT-PRIMO clinical practice ePortal to increase smoker engagement with online cessation interventions: a national hybrid type 2 implementation study

**DOI:** 10.1186/s13012-015-0336-8

**Published:** 2015-11-02

**Authors:** Thomas K. Houston, Rajani S. Sadasivam, Jeroan J. Allison, Arlene S. Ash, Midge N. Ray, Thomas M. English, Timothy P. Hogan, Daniel E. Ford

**Affiliations:** VA eHealth Quality Enhancement Research Initiative and Center for Healthcare Organization and Implementation Research, Bedford VA Medical Center, Bedford, MA USA; Division of Health Informatics and Implementation Science, Quantitative Health Sciences, University of Massachusetts Medical School, 55 Lake Avenue N, Worcester, MA USA; Department of Health Services Administration, School of Health Professions, University of Alabama at Birmingham, Birmingham, AL USA; Division of General Internal Medicine, Johns Hopkins School of Medicine, Baltimore, MD USA

**Keywords:** Smoking cessation, Web-assisted tobacco intervention, Implementation science, E-referrals, Public health informatics, Medical practice

## Abstract

**Background:**

Effective web-assisted tobacco interventions (WATIs) have been underutilized by smokers; moreover, despite practice guideline recommendations, clinical teams do not routinely refer smokers to WATIs. Our goal was to test a clinical practice innovation, an ePortal designed to change practice and patient behavior. Our hypotheses were that the integrated system would result in increased smoker referrals, with an automated follow-up system resulting in more smoker registrations and finally augmentations of the WATI would result in more smokers quitting at 6 months.

**Methods:**

*Practice ePortal Implementation Trial*: Practices (*n* = 174) were randomized to an online practice ePortal with an “e-referral tool” to the WATI (e-referred smokers received automated email reminders from the practice) and with practice feedback reports with patient tracking and practice-to-patient secure messaging versus comparison (a paper “referral prescription”). Implementation success was measured by the number of smokers referred and smokers registering.

*Clinical Effectiveness Trial*: To estimate the effectiveness of the WATI components on 6-month smoking cessation, registered smokers were randomized into three groups: a state-of-the-art tailored WATI control [control], the WATI enhanced with proactive, pushed tailored email motivational messaging (messaging), and the WATI with messaging further enhanced with personal secure messaging with a tobacco treatment specialist and an online support group (personalized).

**Results:**

*Practice ePortal Trial results*: A total of 4789 smokers were referred. The mean smokers referred per practice was not statistically different by group (ePortal 24.89 (SD 22.29) versus comparison 30.15 (SD 25.45), *p* = 0.15). The e-referral portal implementation program resulted in nearly triple the rate of smoker registration (31 % of all smokers referred registered online) versus comparison (11 %, *p* < 0.001).

*Clinical Effectiveness Trial results*: Active smokers randomized to the personalized group had a 6-month cessation rate of 25.2 %, compared with the messaging group (26.7 %) and the control (17 %). Next, when using an inverse probability weighted selection model to account for attrition, those randomized to the two groups that received motivational messaging (messaging or personalized) were more likely to quit than those in the control (*p* = 0.04).

**Conclusions:**

Among all smokers referred, the e-referral resulted in nearly threefold greater registrants (31 %) than paper (11 %). The practice ePortal smokers received multiple reminders (increasing registration opportunities), and the practices could track patient progress. The result was more smokers registering and, thus, more cessation opportunities. Combining the proactive referral and the WATI resulted in higher rates of smoking cessation.

**Trial Registration:**

Web-delivered Provider Intervention for Tobacco Control (QUIT-PRIMO) - a randomized controlled trial: NCT00797628.

**Electronic supplementary material:**

The online version of this article (doi:10.1186/s13012-015-0336-8) contains supplementary material, which is available to authorized users.

## Introduction

Clinical practices have embraced routine screening for tobacco use, [1, 2] brief advice to quit is becoming universal, [3, 4] and pharmaceutical treatments are increasing. In addition to medications and nicotine replacement therapy, behavioral support for quitting is recommended, and clinical practice guidelines recommend practices that refer patients to publicly available resources including telephone quit-lines and web-assisted tobacco interventions [5]. However, clinicians infrequently refer smokers to publicly available resources including web-assisted tobacco interventions (WATIs) [6, 7].

Practice barriers to referral include limited time and competing demands, [8] lack of a champion, staff training, as well as motivation [9]. Most WATIs have not been engineered to connect with clinical practices or provide post-referral feedback on their patients’ progress online. Innovative implementation trials are needed to demonstrate the effectiveness of integrated clinical and technology-assisted tobacco interventions [6, 10]. As defined by Curran et al., hybrid type 2 trials include dual testing of the implementation (practice ePortal intervention) and the clinical intervention (the WATI in this study) [11]. These hybrid implementation-effectiveness studies are appropriate (1) when there has been gathering evidence for clinical efficacy (creating “implementation momentum”), but further detailed effectiveness evidence is needed, and (2) when an implementation strategy addressing barriers to implementing the clinical intervention (the WATI) has not been fully tested in a real-world practice setting.

Questions remain about how to best help clinical practices help their smokers avail themselves of technology-assisted interventions, such as WATIs. Paper-referral “information prescriptions,” brochures with information about web services, have been recommended to encourage patients to use online resources such as Medline Plus [12] with limited evidence to support implementation. These information prescription brochures are a relatively passive form of recruitment and may be forgotten when the patient returns to their home. Our prior work [13, 14] has demonstrated the potential of an ePortal with an e-referral tool that creates automated reminders to patients, sent by email, with personal advice from their physician encouraging use of the WATI. These “e-referrals” represent a more active referral, a sort of warm handoff of the smoker from the clinical practice to the automated WATI. The ePortal innovation also includes tools designed to provide positive feedback to practices—practices can track registration and cessation progress of their patients and send individual doctor-to-patient secure messages to encourage participation. In pilot tests, clinical practices (*n* = 6) assisted in improving the portal interface [9]. The providers liked the simplicity and ease of the referral process, including the system that automatically emailed patients to remind them to visit the site. The providers were enthusiastic about the practice reports, expressing that the nationwide comparison of referrals per practice and the tracking of their patient panel could serve as motivation to improve. The providers were enthusiastic about the potential of secure messaging to engage patients in their own care but provided several recommendations for improvement and noted potential barriers and facilitators (Table 1). However, testing the ePortal concept in a large implementation trial has not yet been reported.

**Table 1:** Implementation facilitators and barriers, with resolution to barriers

Identified barriers and facilitators to implementation			
Issue	Barrier	Facilitator	Resolution
Ease of system use		X	
Perceived potential to affect care		X	
Difficulty contacting the practice and lack of study champion	X		Each practice was requested to identify two staff members to serve as implementation coordinators to be the primary contacts for the practice and would work with our study personnel
Lack of training and registration difficulties	X		• Created a proactive helpdesk to enhance our study personnel’s availability for technical assistance.
			• Provide training to implementation coordinators in the referral intervention and act as trainers for other staff
			• Supported the referral process throughout the six months.
Lack of motivation and start-up incentives	X		Increased both extrinsic (E) and intrinsic (I) motivation
			• Financial incentive for participation in initial training session (E)
			• Motivational interviewing into each interaction (I)
Forgetting to refer	X		• Called implementation coordinators to aid them in the registration process and answer questions
			• Training calls included experiential hands-on practice with referrals (using simulated “test” patients).
			• Increased work-flow support (see helpdesk)
			• Included the printed information prescription pads to use simultaneously with online referral
			• Developed posters to serve as visual stimulation to use the system and to encourage patients to talk with their provider about quitting
			• Provided a 1-page instruction sheet outlining the steps for referring patients were sent to practices

Currently, the public health potential impact of the WATIs has not been recognized due to limited participation by smokers, [15] in part related to the challenges of clinical practice implementation of referrals and integration of the WATI in clinical care. We designed an implementation program to support ePortal implementation in clinical practices. Although challenging, trials’ testing implementation success and clinical effectiveness are increasingly valued in implementation science as many of our clinical interventions are both guidelines recommended and in need of further effectiveness data. Thus, in the “Quality Improvement in Tobacco-Provider Referrals and Internet-Delivered Microsystem Optimization (QUIT-PRIMO)” trial [14, 16], we randomized 174 US community-based primary care practices and now report the implementation success of the ePortal at the practice level and the subsequent effect of the WATI on 6-month point prevalence abstinence at the patient level.

As conceptualized, the integrated intervention is designed to have a sequence of effects on the process of care within each clinic, with the potential to influence provider behavior (nurses and physicians), processes of care, and patient behavior. Thus, we have designed our main evaluation to assess key areas of influence, which we have abbreviated as Refer-Go-Quit. At the practice level, we hypothesize that more patients will be referred with the ePortal (hypothesis 1: Refer) and that the proportion of referred patients who go to the WATI due to the ePortal will be greater compared with a paper-referral control (hypothesis 2: Go). Among the patients registered, the proportion of smokers who quit at 6 months will be greater among those in an augmented WATI, compared with a standard interactive WATI (hypothesis 3). As described, we augmented a standard WATI with proactive motivational messages (messaging arm) and with personalized access to a tobacco treatment specialist and online support group (personalized arm). Below, we detail the methods of the practice-level implementation study (trial 1) and the patient-level behavior change intervention (trial 2).

## Methods

We conducted a hybrid type 2 implementation randomized trial (ClinicalTrials.gov #NCT00797628) with two levels of randomization. Effectiveness evidence for the WATI is increasing [11, 14–16]. Common tools for smokers include tailored motivational materials and interactive decision aids, assessing perceived barriers and benefits and providing feedback. Although referral to the WATI is recommended in the current practice guidelines for smoking cessation, more clinical effectiveness data is needed, especially comparing individual components of effective interventions. A recent Cochrane review of WATIs found considerable heterogeneity of effect. We specifically are measuring both the relative implementation success of two practice-level implementation strategies (a paper referral versus a practice portal with e-referral) and the comparative effectiveness of varying intensities of the patient WATI [17]. The trial protocol was published in *Implementation Science*, and pilot testing of the ePortal has also been published [14, 16].

**Fig. 1:**
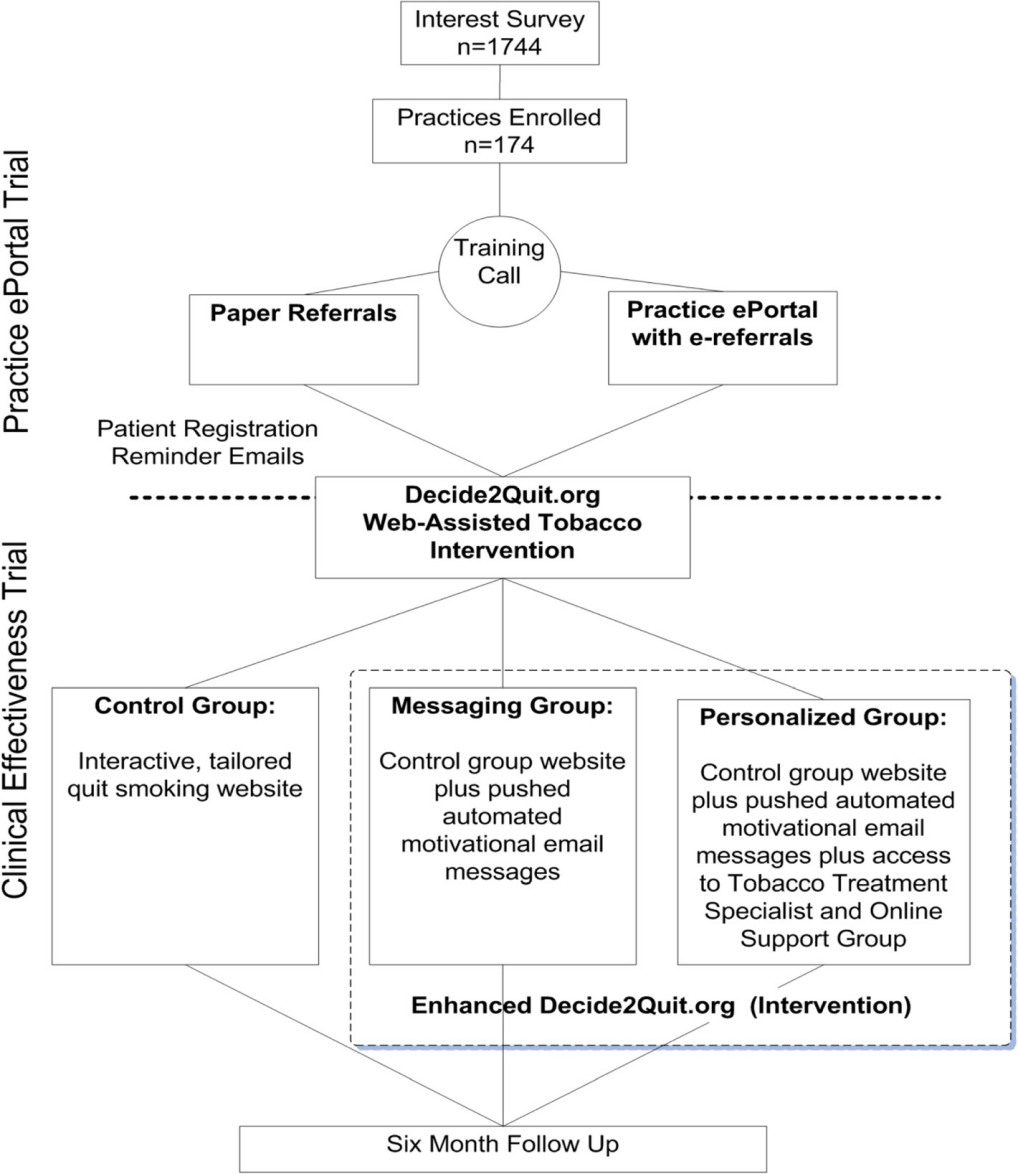
Practice ePortal (trial 1) and clinical effectiveness (trial 2) Randomization Flow

In overview, we first randomized 174 community-based practices; half of the practices (the comparison) implemented paper referrals to encourage patient WATI use, and the other half (the practice intervention) used an innovative online practice ePortal with an “e-referral tool” to the WATI (e-referred smokers received emails encouraging them to register). To test the comparative effectiveness of WATI features, we then randomized the registered smokers to receive standard or enhanced features (Fig. 1). In addition to the screenshots of the interventions included in the Additional files, we have also provided brief mpeg movies as Additional files 2 and 4 so that readers can best understand the practice and patient web systems. To best appreciate the facets of the two trials, we report the methods of the practice implementation trial and analyses and the patient effectiveness trial and analyses separately and follow with the results.

## Trial 1: clinical practice ePortal implementation methods

As discussed, the primary research aim for trial 1 was to measure rates of smoker referral and rates of smoker registration, comparing the ePortal and paper-referral implementation strategies. We hypothesize that more patients will be referred with the ePortal (hypothesis 1: Refer) and that the proportion of referred patients who go to the WATI due to the ePortal will be greater compared with a paper-referral control (hypothesis 2: Go). Practice randomization began in June 2011. Practices were randomized at the practice level to paper referral or to the practice ePortal with e-referrals. Allocation was generated from a prepared randomization table (a randomization table with blocks of 10) and was automated by the system during the registration of the first provider at the practice. All others who registered from that practice were then randomized to the same arm (paper or e-referrals). We followed practices for 6 months from their initial contact. The study was approved by the Institutional Review Board, and the protocol was overseen by a data safety and monitoring board.

### Setting (clinical practices)

Community-based (non-university) US general, family, or internal medicine practices were eligible. We used a mailing list of practices and recruited until we achieved 174 practices. Practices implementing the paper- or e-referral implementation strategies chose which smokers to refer. During training, the practices were encouraged to refer smokers regardless of whether they were ready to quit smoking. All smokers in these practices were eligible for referral. Using intent to treat, all active smokers who registered (including online consent procedure) were included in a 6-month follow-up, regardless of the level of activity on the WATI.

### Practice-level comparison versus ePortal practice intervention

#### Paper referrals

Paper-referral practices were provided preprinted pads of “information prescriptions” with their office information, a space for the provider to sign, and the WATI for smokers website address (www.decide2quit.org [D2Q]) [16]. The paper referrals were provided to patients during their visit.

#### Practice intervention: practice ePortal with e-referrals, feedback, patient tracking, and messaging

Practices in the ePortal arm were also provided the paper “information prescriptions” to facilitate conversations with patients, but further had access to our practice ePortal (see screenshots in Additional file 1 and accompanying video, Additional file 2—entitled “ReferaSmoker.org”). The core feature of the practice portal was a secure user-friendly form where providers could refer smokers to D2Q by entering their email addresses [9, 16, 18]. The e-referral created an identity link (patient email) with D2Q, to send reminders of the referral, and also created a practice link, connecting patient and practice. After e-referral, the smokers received up to 10 reminder emails encouraging D2Q registration. Within the practice e-referral portal, the practice link allowed us to provide clinical practices with real-time dashboard monitoring about their practice’s smokers’ registration and progress in the WATI. Each time a registered smoker logged in, the WATI collected the smokers’ quit status which was then fed back to the clinical practice dashboard. Through the ePortal, practices could also send additional personalized motivational messages to their patients. The practice ePortal also provided each practice with performance feedback reports, comparing the number of referrals in their practices with all participating e-referring practices.

### Implementation facilitation in both comparison and intervention practices

Published in detail elsewhere, [14] a facilitation-based program, guided by the Promoting Action on Research Implementation in Health Services (PARIHS) framework, was instituted to support implementation referrals in both the paper and practice portal arms. PARIHS provides a conceptual framework that incorporates various influences that interact within implementation. When used as a guiding framework, PARIHS can highlight important evidence and contextual factors that may influence an implementation effort and can inform the development of a suitable facilitation-based implementation strategy.

Our facilitation program included individualized telephone/Internet trainings using an academic detailing approach. In our pilot test, staff turnover was identified as a challenge to sustained implementation, so each practice was asked to identify two implementation coordinators (physicians, nurses, or other staff). These two implementation coordinators acted as points of contact and champions. They were trained in the referral intervention and acted as train-the-trainers providing information to the rest of the practice and encouraging adoption. Training was tailored to randomization arm (with paper-referral practices being trained on the paper referral only and provided an overview of the Decide2Quit.org WATI). For clinical teams randomized to the practice portal, training included hands-on demonstrations of the ReferASmoker.org website, including initial registration, practice e-referring a “test” smoker, and exploring Decide2Quit.org. As reminders, for both arms, a series of four motivational booster emails were sent over a 6-week period. For ongoing facilitation, our study team completed a total of six proactive booster facilitation calls (approximately 15–30 min) assessing perceived barriers, strategizing solutions, and reinforcing success. Table 1 outlines our changes based on our feedback to facilitate the implementation of the referral.

Our implementation was designed to have several pragmatic aspects [19] including that we provided a brief one-time practice facilitation training online about referral procedures for all practices (both paper and e-referrals), encouraged referrals through motivational follow-up calls, but did not conduct in-person detailing, nor did we incentivize practices for each referral. Each practice was allowed to consider how to best integrate the referrals into their workflow, [14] allowing variation in implementation fidelity. Consistent with the goals of an implementation science study, we did not have strict guidelines on who to refer or the number of referrals required of practices, nor did we provide referrals incentives. The practices were told that the web-assisted tobacco intervention had content for all smokers, regardless of their readiness to quit. We provided them the system and training, but the adoption and use of the system was left to them. Thus, we anticipated and measured variation in implementation success.

### Trial 1 (implementation) statistical analyses

Baseline practice characteristics were collected through a paper survey as part of the recruitment process. Among the e-referral practices, we measured the use of the components of the portal (referrals, patient secure messaging, practice feedback). For both comparison and intervention practices, we monitored rates of referral and patient registration.

Practice ePortal and e-referral system use data which was collected by the web server as activity logs linked to each individual practice. Self-report referral data was also collected for all paper- and e-referral practices during follow-up booster calls. Additionally, referral data for the e-referral practices was also tracked online (self-report and web-tracked referral rates were highly correlated (coefficient = 0.7)). To avoid bias from differential measurement, self-report of referral was used as the primary measure across both groups. For practice-level implementation success analyses, our outcomes were the number of reported referrals per practice and the number and proportion of referred patients who registered per practice. We analyzed using both *t* test and negative binomial models as appropriate for count data with over-dispersion.

## Trial 2: clinical effectiveness of the web-assisted tobacco intervention

Patients from both paper-referral and e-referral practices had access to the D2Q WATI registration. D2Q was designed with separate modules, allowing patient-level randomization. Once patients (from all practices) registered and completed an online consent form, the WATI system automatically randomized them to three levels of the WATI. A randomization table, block randomized with blocks of 10, was pre-populated, and the system used this table to assign different features of the WATI to different smokers. As technology-assisted interventions are often multi-component and evolve over time, and fractional factorial designs like ours are increasingly used to assess the effect of various components [20–22]. These designs are more efficient than full factorial designs. As all smokers received some level of WATI, they were blinded to the assignment group. Investigative team members involved in 6-month smoking cessation outcomes (7-day point prevalence measure) were blinded to randomization assignment.

### Patient sample

Inclusion criteria included being an active smoker, having been referred from a paper-referral or e-referral practice, and completing consent procedure. An active smoker was defined as smoking at least one puff of a cigarette or cigar in the last 7 days. We included smokers regardless of whether they were ready to quit or not yet ready. All active smokers 18 and over were included. As we did not require a certain level of participation, all smokers randomized were considered for intent-to-treat analyses.

### Intervention: Decide2Quit.org web-assisted tobacco modules

The D2Q WATI was designed for all smokers, supporting cessation-induction for those not ready to quit and acting as an aid-to-cessation for those preparing to quit [16]. D2Q was implemented as an adaptable service with modules that could be engaged based on assigned group. Thus, within practices, individual smokers were allocated, using our online system and a block-randomization table similar to the practice level to one of the three increasingly enhanced versions of D2Q. The smokers completed an online consent form prior to randomization. The interventions received by the three randomized groups of smokers are described below.

#### The control

The smokers randomized to the active control received *an interactive, tailored quit smoking website*. This module included motivational information tailored to readiness to quit (not thinking of quitting, thinking of quitting, preparing to quit) and interactive risk, decisional balance, and cessation barrier calculators and games linking the chemicals in smoking with their other uses (e.g., formaldehyde is used in both cigarettes and in embalming). The control also included a library of informational resources about smoking and sections on seeking social support and talking to your doctor about quitting (see screenshots in Additional file 3, and accompanying video, Additional file 4). These tools are variations of those in many previously studied evidence-based WATI. As noted in the Cochrane review, WATIs that include tailored content are more likely to demonstrate an effect [17]. Thus, ours is an active control representing the state of the art in public health websites.

#### The messaging group: pushed motivational email messages module plus control module

For this group, we enhanced the control with a pushed motivational email messaging system. Brief motivational email messages (Additional file 5) were further tailored to an individual smoker’s readiness to quit (not ready to quit, thinking about quitting, preparing to quit, actively quitting). In the first week of registration, four email messages were sent to the smoker, followed thereafter by two email messages per week. To enhance the personal relevance of the messages, our motivational email messaging system included messages written by smokers for other smokers [23].

#### Personalized group: personal support module, messages module, and control module

In addition to the above functions, the personal support module further included the following: (1) an innovative secure messaging portal allowing asynchronous electronic communication between smokers and trained tobacco treatment specialists (TTS) hired to participate in the intervention team and (2) a link to an online support group (BecomeAnEx.org).

Although trained at two training centers, all TTS were trained for the following: determinants of nicotine dependence; pharmacotherapy; counseling theory and practice, including motivational interviewing, treatment strategies, substance abuse, and mental health disorders; and intake and treatment planning. Each TTS was required to pass a TTS exam as completion of the course. All TTS met weekly prior to recruitment of the first participants, and the meetings continued for several weeks into the study. During these meetings, the TTS reviewed the principles of motivational interviewing. Once recruitment began, the TTS met to discuss any thoughts, issues, or challenges in expressing Motivational Interviewing skills via asynchronous secure messaging.

Asynchronous communication between patients and TTS began with a message sent by the patient once they logged into the site. The TTS have access to a portal in which they checked daily for any assigned messages. The TTS created responses based on current guidelines and using Motivational Interviewing techniques [24]. Examples include rolling with resistance, asking patients to identify barriers, evaluate self-efficacy, and determining level of addiction.

One such example message sent and a reply correspondence is the following:

Patient: Hi. My doctor said he wanted me to check out this website. I love smoking, but I know I should quit, but I’m having trouble finding incentive. Can you help?

Response: Hi. You’ve made a great step in the right direction by logging in to the website. I know it can be challenging to begin to think about quitting. You mentioned having trouble finding incentives…..what about making finding incentives a first step/goal? Make a list of all the reasons you have to quit. Then I’m sure the motivation will follow. Also, you could think about what you think will be your biggest barriers to successfully quitting. Identifying what sorts of things keep us from quitting is the first step in overcoming them! Message me back and let me know what you think!

Note that in our original protocol, we planned to randomize the smokers into two groups, the control and personalized. During the progress of this 5-year trial, evidence for web-assisted tobacco interventions continued to develop, especially regarding the importance of tailored messaging. Our study leadership recognized the importance of differentiating the messaging intervention and the personalized engagement with a tobacco treatment specialist online. Thus, the original control group was further randomly subdivided into the control website-only group and the messaging group (as described above). The reason for splitting the original groups into control, messaging and personalized groups, an augmentation of the original protocol, was that the literature on tailored messaging has continued to evolve, and our team decided it would be appropriate to compare messaging to personalized and messaging to control.

### Patient characteristics and outcomes measurement

The smoker characteristics and D2Q system use were collected online. At 6 months, a follow-up was conducted using both online and telephone surveys to collect a 6-month and 7-day point prevalence self-reported cessation.

At the patient level, we hypothesized that the proportion of smokers who quit at 6 months would be greater among those in the fully enhanced “personalized” group as compared with the messaging group and the standard control only. We also hypothesized that the proportion of smokers who quit at 6 months would be greater among those who received motivational messages (personalized and messaging combined), compared with the control.

### Statistical analysis of a 6-month and 7-day point prevalent cessation

Our analyses were framed around the hypotheses and used intent-to-treat principles. The smokers were analyzed as randomized regardless of the level of engagement with the systems (fidelity of intervention). All analyses were conducted in the STATA statistical software package (StataCorp. 2011. Stata Statistical Software: Release 12. College Station, TX: StataCorp LP) with confirmation in SAS (SAS software, Version [8] of the SAS System for Windows).

For our primary clinical effectiveness outcome (6-month and 7-day point prevalence cessation), we compared those randomized to receive all enhanced features (personalized) with the messaging Group and then the control WATI. To analyze the effect of the messaging module, we compared the control with all those who received the messaging module (the messaging group and the personalized group combined).

A challenge for all cessation-induction trials is missing outcome data [25–27]. This is especially true in light-touch technology-assisted interventions. Most often, a complete case or missing case (penalized imputation) indicative of smoking analysis has been implemented in logistic regression. However, the reason that we did not use penalized imputation as the primary outcome is that new literature has been published suggesting that penalized imputation (or missing = smoking) is not a conservative approach and can be biased toward the intervention [26]. Thus, for studies with missing cessation data greater than 10–20 %, experts recommend using selection models to examine the robustness of findings [25]. We compared the characteristics of the patients who remained in the trial and those lost to follow-up (see Additional file 6). For our primary models, using a generalized linear model with a logit link to evaluate cessation outcomes and GEE methods to account for clustering within practices, we implemented a selection model using inverse probability weighting to determine the potential effect of the missing data. First, based on covariates available within the dataset, we developed a logistic regression model to predict the amount of missing data. Then, we calculated the inverse probability of not being missing and weighted the main analysis by this probability [28]. Since the primary models each examine a hypothesis (and secondary completed cases and penalized imputation models are confirmatory and not exploratory), we report each *p* value with no adjustment for the total number of tests conducted.

## Results

Following the format of the “Methods” section, we first present the practice (ePortal) implementation trial success (trial 1) and then the clinical effectiveness (Trial 2) of the WATI on 6-month 7-day point prevalent cessation.

## Trial 1: clinical practice ePortal implementation

One thousand, seven hundred and forty-four primary care practices expressed interest in participating, and 174 (10 %) fully completed enrollment and randomization. Those who participated were smaller (65.1 % solo practice versus 55.6 % in non-participating, chi-square *p* = 0.02) and somewhat more likely to report previous referrals of smokers to quit-lines (47.3 % in participating versus 39.9 % in non-participating, chi-square *p* = 0.06) and more frequently to report seeing over 20 smokers per week (71 % versus 65 %, chi-square *p* = 0.1).

The 174 practices who participated were small (solo physician = 68 % in e-referral, 63 % in paper-referral) and saw a median of 40 smokers per week (Table 2). The volume of patients was balanced across e-referral and paper-referral practices. All practices had at least one staff trained; 67.8 % of the e-referral practices and 72.4 % of the paper-referral practices had two; 8.1 % of e-referral practices and 4.6 % of paper-referral had an additional member of the practices trained. Of those trained, 17 % were physicians, 4 % nurse practitioners/physician assistants, 10 % nurses, and 69 % medical assistants or other office staff.

**Table 2:** Trial 1: comparing paper-referral and ePortal practice implementation, characteristics of 174 community-based practices

	E-referral (*N* = 87)		Paper-referral (*N* = 87)	
	*N*	%	*N*	%
Type				
Internal medicine	36	41.4	44	51.2
Family medicine	50	57.5	39	45.3
General practice	1	1.1	3	3.5
Number of physicians				
1	59	68.6	54	62.8
2	15	17.4	23	26.7
3 or more	12	14.0	9	10.5
Region of country				
Northern East	21	24.1	21	24.1
South	32	36.8	36	41.4
Middle West	16	18.4	19	21.8
West	18	20.7	11	12.6
	E-referral		Paper-referral	
	*N*	Median (IQR)	*N*	Median (IQR)
Number of patient visits/week	84	123 (100–188)	87	120 (90–160)
Number of smokers/week	87	40.0 (20–60)	87	40.0 (20–50)
Number of computers	84	5.1 (4–9)	85	4.1 (3–7)

*N*s vary between 84 and 87 due to the small amount of missing baseline data for some variables. All characteristics are not significantly different (*p* > 0.05), comparing e-referral and paper-referral practices

### Use of the ePortal in intervention practices

**Fig. 2:**
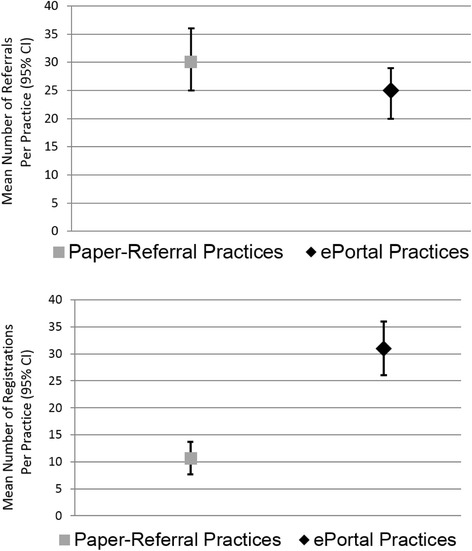
Trial 1: comparing paper-referral and ePortal practice implementation results (patient referrals to the web-assisted tobacco intervention and subsequent patient registration) among 174 practices. Mean number of referral Per practice (95 % confidence interval) and mean percentage referred smokers who registered (95 % confidence interval)

Eighty-one of the eighty-seven practices randomized to e-referral referred at least one smoker. The total reported number of smokers e-referred within 6 months was 2166 (mean referrals per practice 24.89 (SD 22.29), range from 0 to 142 (interquartile range 8–37)) (Fig. 2). In addition to the automated e-referral driven reminder emails by the system, physicians and office staff from slightly over half of e-referral practices (55 %, 48/87) used the ePortal to send personalized messages to their smokers after registration (among those using messaging, mean messages per practice = 8.7 (SD 15.7), range 1 to 88).

Among the 87 intervention practices, the mean number of views of the practice feedback dashboard was 20.3 ([SD 22.7], range 1 to 130, median 12, interquartile range (IQR) 29.5). To estimate the impact of the practice feedback on referral behavior, we assessed the rates of e-referral stratified by the use of the practice feedback dashboard. E-referral practices that did not use the practice feedback had a mean referral rate of 15 (SD 15) within 6 months, and those that viewed the practice feedback report between 1 and 10 times had a mean of 21 referrals (SD 18), compared with those who viewed them over 10 times (mean 30 referrals [SD 25]) *p* for trend across categories = 0.008.

### Comparing referrals and registrations by randomization (paper versus ePortal)

*Hypothesis 1 (Refer):* Similar to ePortal practices, 82 paper-referral practices had at least 1 referral, and paper-referral practices reported referring 2623 smokers (mean referrals per practice 30.15 [SD 25.45]) in 6 months (not statistically different compared with the ePortal [p = 0.15 for the difference mean referrals] ). The range of paper-referrals was 0 to 150 (IQR 12–42). While the total number of e-referrals was only 83 % as great as the number of paper-referrals, this difference was again not significant based on a negative binomial count regression model (incidence rate ratio = 0.83 (95 % confidence interval (CI) 0.63, 1.08), *p* = 0.17).

*Hypothesis 2 (Go)—smoker registration*: Among ePortal practices, the mean percent of referred smokers registering was 31 % (SD = 25), compared with 11 % (SD 15) from paper-referral practices (*p* = 0.001), as shown in Fig. 2. The mean number registered per e-referred practice was = 6.90 (SD = 8.65) versus 2.85 (SD = 3.45) from the paper-referrals (*p* = 0.01). In further analyses using count regression, during the 6 months of follow-up, e-referred patients registered at 2.42 times the rate of patients referred with paper referrals (incident rate ratio = 2.42, 95 % CI: 1.72–3.40).

**Fig. 3:**
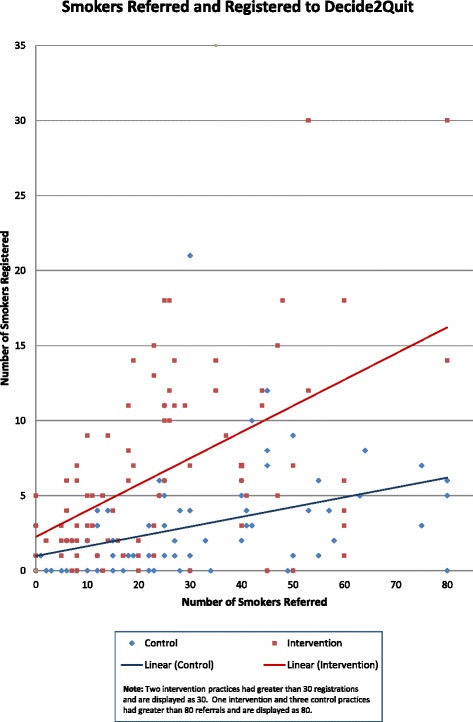
The number of smokers referred and registered by each practice

Figure 3 is a scatterplot that has, on the horizontal access, the number of smokers referred by each practice and, on the vertical axis, the number of smokers registered. Each practice is represented as a point (red for intervention, blue for control), with a super-imposed trend line. Although the dots are similarly distributed across the horizontal axis, there was considerable variation overall in rates of referral. The trend lines show very clearly that you get more people registered per person referred with the ePortal intervention. Note that there were only two control practices that had over 10 smokers register, whereas 22 ePortal practices had over 10 smokers register.

## Trial 2: clinical effectiveness of the web-assisted tobacco intervention

**Fig. 4:**
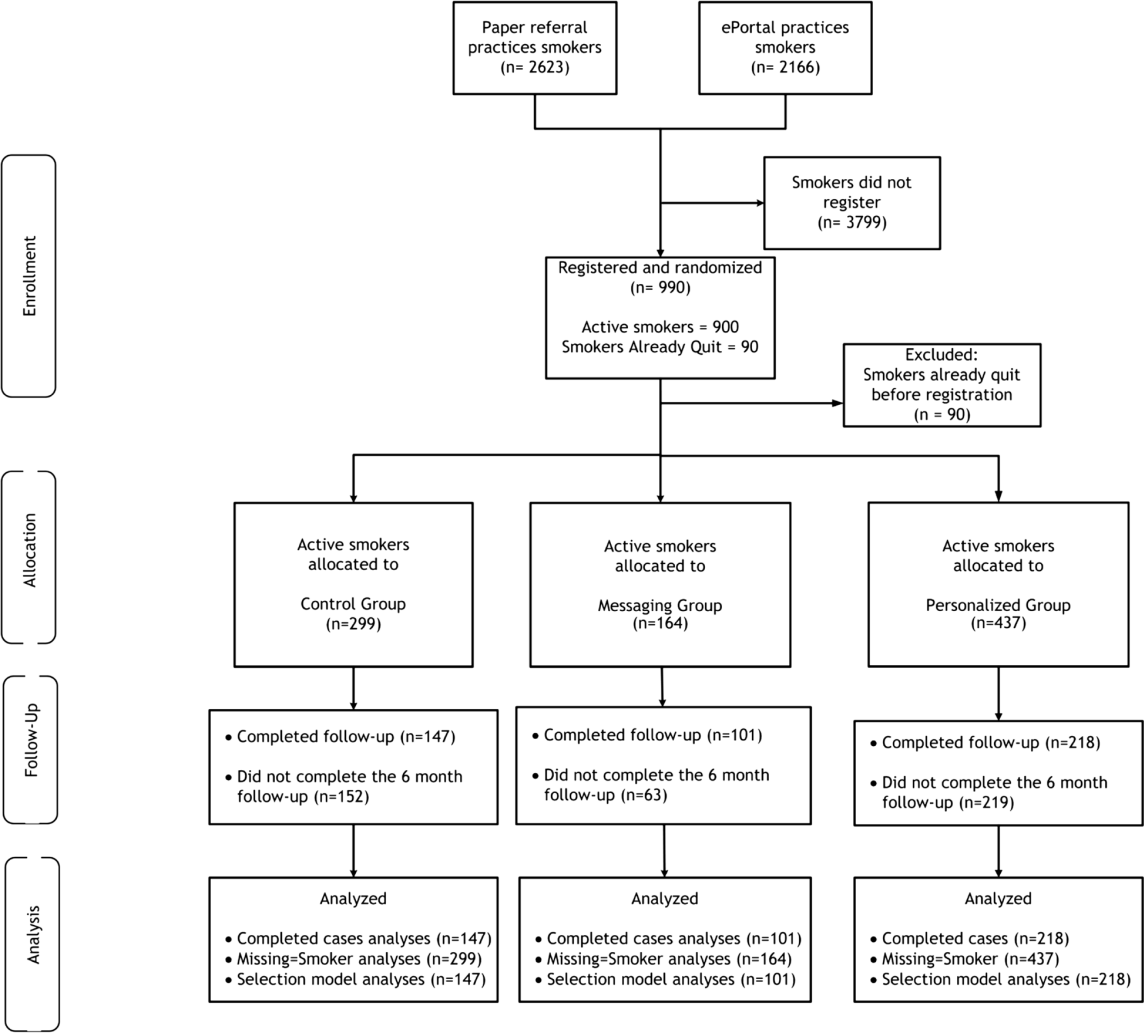
Trial 2: clinical effectiveness of the web-assisted tobacco intervention, consort Diagram

A total of 990 primary care patients registered, with 900 reporting active smoking (see consort diagram—Fig. 4). Most were female (63 %), between 35–55 years of age (50 %), and smoked 11–20 cigarettes per day (Table 3), and these characteristics did not differ by randomization group.

**Table 3:** Trial 2: clinical effectiveness of the WATI, characteristics of active smokers registering from clinical practices^a, b^

	Total		Control		Messaging		Personalized	
	*N*	%	*N*	%	*N*	%	*N*	%
Patient Sex								
Female	570	63	185	62	104	63	281	64
Male	330	37	114	38	60	37	156	36
Patient Age								
19–34	152	17	47	16	27	16	78	18
35–55	454	50	155	52	77	47	222	51
55–64	224	25	71	24	48	29	105	24
65+	70	8	26	9	12	7	32	7
Patient race								
White	769	85	253	85	144	88	372	85
Black or African American	87	10	33	11	14	9	40	9
Others	44	5	13	4	6	4	25	6
Patient education								
Less than high school	75	8	24	8	15	9	36	8
High school graduate	270	30	88	30	41	25	141	33
Some college	380	43	139	47	71	44	170	39
College graduate or more	167	19	46	15	35	22	86	20
Readiness to quit ^c^								
Not thinking of quitting	34	4	13	4	4	2	17	4
Thinking of quitting	699	82	246	83	138	84	315	81
Set a quit date	117	14	39	13	22	13	56	14
Allow smoking at home								
No	460	51	146	49	77	47	237	54
Yes	440	49	153	51	87	53	200	46
Number of cigarettes per day								
0–10	241	27	84	28	42	26	115	26
11–20	460	51	149	50	81	49	230	53
>20	199	22	66	22	41	25	92	21
Visited other smoking cessation websites before								
No	782	87	264	88	142	87	376	86
Yes	118	13	35	12	22	13	61	14
Quit attempt (1 day or more) in past 12 months								
No	424	47	148	50	71	43	205	47
Yes	476	53	151	51	93	57	232	53

^a^ Nine hundred ninety patients registered, of whom only 900 were current smokers (see Fig. 3). ^b^ Characteristics were not significantly different between randomized groups (control, messaging, personalized). ^c^ Denominator 850 for this variable, as 50 patients did not complete status at initial registration

### Use of the web-assisted tobacco intervention (Decide2Quit.org) by smokers

The number of return visits to the WATI differed by treatment condition and was highest in the personalized group (mean visits 2.52 (SD = 4.09), median 1, IQR 2), followed by the messaging group (1.81 (SD = 1.26), median 1, IQR 1), and followed by the control “state-of-the art WATI” (mean 1.71 (SD = 1.14), median 1, IQR 2) (control versus messaging, *p* = 0.37; control versus personalized, *p* < 0.001; trend across groups, *p* < 0.001). In the personalized group, the smokers had access to a tobacco treatment specialist and online peer support group; 33.64 % (*N* =147) of smokers used the asynchronous tobacco treatment specialist messaging (mean 2.31 messages (SD = 4.29), median 1, IQR 1), and 21.74 % (*N* = 95) used the online peer support group (mean 1.78 visits (SD = 1.52), median 1, IQR 1).

### Six-month and seven-day point prevalence smoking cessation (patient hypotheses)

Of the 900 active smokers, 14 % declined follow-up, and 34 % could not be contacted (Fig. 4). Smoking cessation (Tables 4) was higher (25 % cessation in completed cases) among those in the fully enhanced (personalized) compared to those randomized to standard functions (control) (17 %) (*p* = 0.06), although this is not statistically significant. The personalized group had a similar rate of cessation to those in the messaging group (25.23 versus 26.73 %), which was not significantly different. Using the selection model (Table 4), the smokers in the enhanced groups who received pushed motivational messages (personalized and messaging) were more likely to quit (odds ratio 1.69 (95 % CI 1.03–2.8) *p* = 0.038) as compared with the control-without-messages group. In secondary analysis with all those lost to follow-up assigned to missing, we again found a similar result (odds ratio 1.732, (95 % CI 1.08–2.77) *p* = 0.022) . When comparing across the three modeling techniques, note that there was no meaningful nor significant difference comparing the messaging group to the personalized group, all comparisons of personalized versus control did not reach *p* = 0.05 (*p* value range 0.063 to 0.072, with odds ratios from 1.578 to 1.660), and all comparisons of those receiving messages did reach significance (*p* value range from 0.021 to 0.038 and odds ratios 1.688 = 1.732).

**Table 4:** Trial 2: Clinical Effectiveness of the WATI, Six-month cessation by allocation to Technology-Assisted Tobacco Intervention features

**Comparing Control, Messaging, and Personalized groups** ^**a**^ **; Point estimates and Odds Ratios**					
		(CONTROL) Standard Tailored Website	Messaging group	Personalized Group (All features)	Personalized versus control
Completed cases	Percent	25/147 (17%)	27/101 (26.7%)	55/218 (25%)	0.063
	Odds Ratio	reference	1.781 (0.962-3.296)	1.647 (0.971-2.791)	0.064
Missing = Smoking	Percent	25/299 (8.4%)	27/164 (16.5%)	55/437 (12.6%)	0.071
	Odds Ratio	reference	2.160 (1.208-3.863)	1.578 (0.959-2.595)	0.072
Selection Model	Odds Ratio	reference	1.790 (1.208-3.863)	1.660 (0.959-2.595)	0.064
**Comparing Control and those Receiving Messages (Messaging or Personalized)**					
		Standard without messages (CONTROL)	With Messages (Groups Messaging or Personalized)		With Messages versus Control (p)
Completed cases	Percent	25/147 (17%)	82/319 (25.7%)		0.038
	Odds Ratio	reference	1.688 (1.026-2.779)		0.039
Missing = Smoking	Percent	25/299 (8.4%)	82/601 (13.6%)		0.021
	Odds Ratio	reference	1.732 (1.081-2.774)		0.022
Selection Model	Odds Ratio	reference	1.699 (1.026-2.813)		0.039
Note: Table 4 uses completed cases, missing equals smoking, and selection modeling methods					

^a^ Smokers received either a standard interactive Internet site (CONTROL), the standard enhanced with pushed automated motivational email messages (MESSAGING), or the features of CONTROL and MESSAGING plus access to secure messaging with a tobacco treatment specialist and a smoker to smoker online support group (PERSONALIZED)

In this trial, we recruited all active smokers, including those in the motivation phase (not ready to quit, thinking about quitting), and precessation phase (preparing to quit, set a quit date). In stratified analyses, the motivational messages were similarly effective among those in the motivation phase (6-month cessation 23 versus 15 % control; odds ratio 1.616, 95 % CI 0.91–2.87) and those who already quit (37 versus 24 % control; odds ratio 1.932, 95 % CI 0.59–6.38).

## Discussion

We successfully completed a large, community-based implementation trial, integrating a provider e-referral system and a patient-directed WATI. Among the smokers referred, the e-referral was far more effective, with nearly threefold greater registrants (31 %) than paper (11 %). Enhancing the standard control with motivational messages improved the comparative effectiveness of the WATI. For the average e-referral clinical practice, the combination of increased patient registration and increased relative cessation of the virtually integrated e-referral/enhanced technology-assisted tobacco intervention resulted in enhanced quality of care for smokers.

The rate of referral was slightly less in ePortal practices. This is likely due to the increase in effort required to engage with the ePortal. In our pre-implementation pilot work, we reported that several physicians believed that an e-referral requires some additional effort from and training of practice staff [9]. With this in mind, we tried to integrate the ePortal into clinic’s workflow. We designed the e-referral portal to use the data collected to provide feedback and positive reinforcement to providers, feedback that was not available to paper-referral practices. Practices could also use the data collected to provide feedback and positive reinforcement to providers, feedback that was not available to paper-referral practices. As noted, more views of the practice reports were associated with more e-referrals. In the initial implementation of the system, the e-referral providers had found these reports helpful in motivating continued e-referral activity, and thus, we integrated performance feedback into the portal [9].

As hypothesized, paper referral was a “thin” intervention, with only 11 % of smokers registering. There are few comparisons for benchmarking our registration rates. Offline, fax referrals have been used as a proactive way to refer patients to telephone quit-lines [10, 29]. Once referred, quit-lines will proactively call patients, and of patients referred, quit-line staff was successful in enrolling approximately 20–30 % [10, 29, 30]. The e-referral registration rate of 31 % is important when considering the “higher-than-usual-for-Internet-interventions” percentage of referred patients who were not ready to quit [31].

Our positive clinical effectiveness result that smokers randomized to receive enhanced, proactive features were more likely to quit extends our knowledge beyond the conclusions of the recent Cochrane review of WATIs [17]. In a recent review of WATI by the Cochrane collaboration, pooled results from trials that compared interactive WATI to usual care or written self-help detected significant effects in favor of the intervention with a calculated odds ratio of 1.46 (and relative risk of 1.41)—based on pooled data from three studies. To place our study into context, note that these WATI trials have compared the WATI with usual care or written materials. Our absolute difference in cessation (ranging from 8.7 to 5.2 % favoring with messages over control) is greater than prior WATI trials, even considering our robust control condition. Our control was an evidence-aligned state-of-the-art website built with extensive tailoring based on readiness to quit and other interactive components. Using this active control, we then added enhanced proactive components (e.g., pushed automated messages). Our results support the recent report on the effectiveness of pushed motivational text messages [32]. These short, frequent messages spaced over time are more likely beneficial than modular “bolus” online motivation.

In the personalized group, the further availability of a tobacco treatment specialist and online support group was minimally utilized and did not result in a marginal improvement in effectiveness. This arm may not have been engaging enough to smokers, resulting in less-than-optimal rates of participation with the TTS. Given the asynchronous pattern of communication, this function may have been difficult for patients to engage with and use. Because of the additional marginal cost in the setting of an interactive technology intervention, health systems should consider carefully how best to use tobacco treatment specialists. Further research is needed on how best to engage smokers and tobacco treatment specialist support online.

Consistent with our goal, we successfully registered smokers who were not yet preparing to quit. This places our study in contrast to most web-assisted tobacco interventions who have only recruited smokers who were preparing to quit or actively quitting [31]. In our prior work, we have demonstrated that recruitment from clinical practices results in more smokers not yet ready to quit, as compared with recruitment through other mechanisms, such as Google advertisements, another important benefit to clinical integration of the WATI. In subset analyses, we demonstrated that these harder to reach, not yet ready to quit smokers, also benefitted from the enhanced, messaging component of the WATI, with higher quit rates in the enhanced (messaging or personalized) compared with the control WATI.

All trials have limitations. As we made the decision to separate into three groups (the original control group was further randomly subdivided into the control website-only group and the messaging group), this reduced our power to detect differences by sub-group. Note that although our retention rate of 52 % at 6 months is higher than many published trials of technology-assisted tobacco interventions, [22, 26, 27] the non-response rate is an important consideration in interpreting our patient-level results. As recommended, [25] we used multiple analytic methods to explore the bias that could be introduced by attrition. Recommendations from the Society for Research on Nicotine and Tobacco on the need for biochemical verification [33] state that the degree of misclassification is moderated by characteristics of the smoking cessation intervention. The more intensive the intervention is, the higher the potential for misclassification due to social desirability. Differential misclassification by randomization group, an even more problematic measurement issue, increases with the intensity difference between groups. In studies similar to ours, misclassification and differential misclassification are expected to be lower, where biochemical verification is not required and can introduce other problems, including refusal to participate [33, 34]. The patients in our sample were highly educated, although compared with the sample derived from Google, our sample is actually less educated [31].

## Conclusion

We have presented evidence of implementation success and comparative effectiveness of an integrated primary care practice e-referral/patient motivational system on smoker engagement and cessation. Healthcare systems should move beyond paper-referral “information prescriptions” only. There will always be a place for paper reminders, but they should be considered in a supportive role. For the increasing numbers of patients who can access the Internet, email or text messages, clinical practice e-referrals will result in higher rates of engagement, enhancing the reach of technology-assisted publically available interventions, and potentially enhancing clinical interventions. Future work should also explore making e-referrals an integral function of electronic health records, where providers will increasingly conduct the work of clinical care. In our pilot implementation, our highest e-referring practice had integrated e-referral into their electronic health record [14]. Although our system was limited to smoking, the concept of electronic referrals could be used for other targeted behaviors and chronic disease self-management systems, a fertile area for further studies.

## Additional files

Additional file 1: Trial 1. Comparing paper-referral and ePortal practice implementation, secure practice ePortal .Screenshot of the homepage of ReferaSmoker.org, an interactive website to assist providers in helping their patients quit smoking. Additional file 2: ReferaSmoker.org Brief mpeg video demonstrating the practice web system, ReferaSmoker.org. Additional file 3: Trial 2. Clinical effectiveness of the web-assisted tobacco intervention, enhanced patient website. Screenshot of the homepage of Decide2Quit.org, a multi-modal, evidence-based smoking cessation induction system available via the Internet. Additional file 4: Decide2Quit.org Brief mpeg video demonstrating the patient web system, Decide2Quit.org. ᅟ Additional file 5: Trial 2. Clinical effectiveness of the web-assisted tobacco intervention, brief motivational messages. Examples of the brief motivational email messages that participants within the Messaging and Personalized groups received. Additional file 6: Trial 2. Clinical effectiveness of the web-assisted show tobacco intervention,characteristics of participants who completed a 6-month follow-up and did not complete a 6-month followup. Table of participant characteristics comparing those who did and did not complete the 6-month follow-up.

## Abbreviations

D2Q: Decide2Quit.org
PARIHS: Promoting Action on Research Implementation in Health Services
QUIT-PRIMO: Quality Improvement in Tobacco-Provider Referrals and Internet-Delivered Microsystem Optimization
TTS: tobacco treatment specialist
WATI: web-assisted tobacco interventions
