# Supplementary material for: Evaluating the QUIT-PRIMO clinical practice ePortal to increase smoker engagement with online cessation interventions: a national hybrid type 2 implementation study
Source: Implement Sci. 2015 Nov 2;10:154. doi: 10.1186/s13012-015-0336-8 (PMC4630887; doi:10.1186/s13012-015-0336-8)

**Appendix A: Trial 1: Comparing Paper-Referral and ePortal Practice Implementation, Secure Practice ePortal**


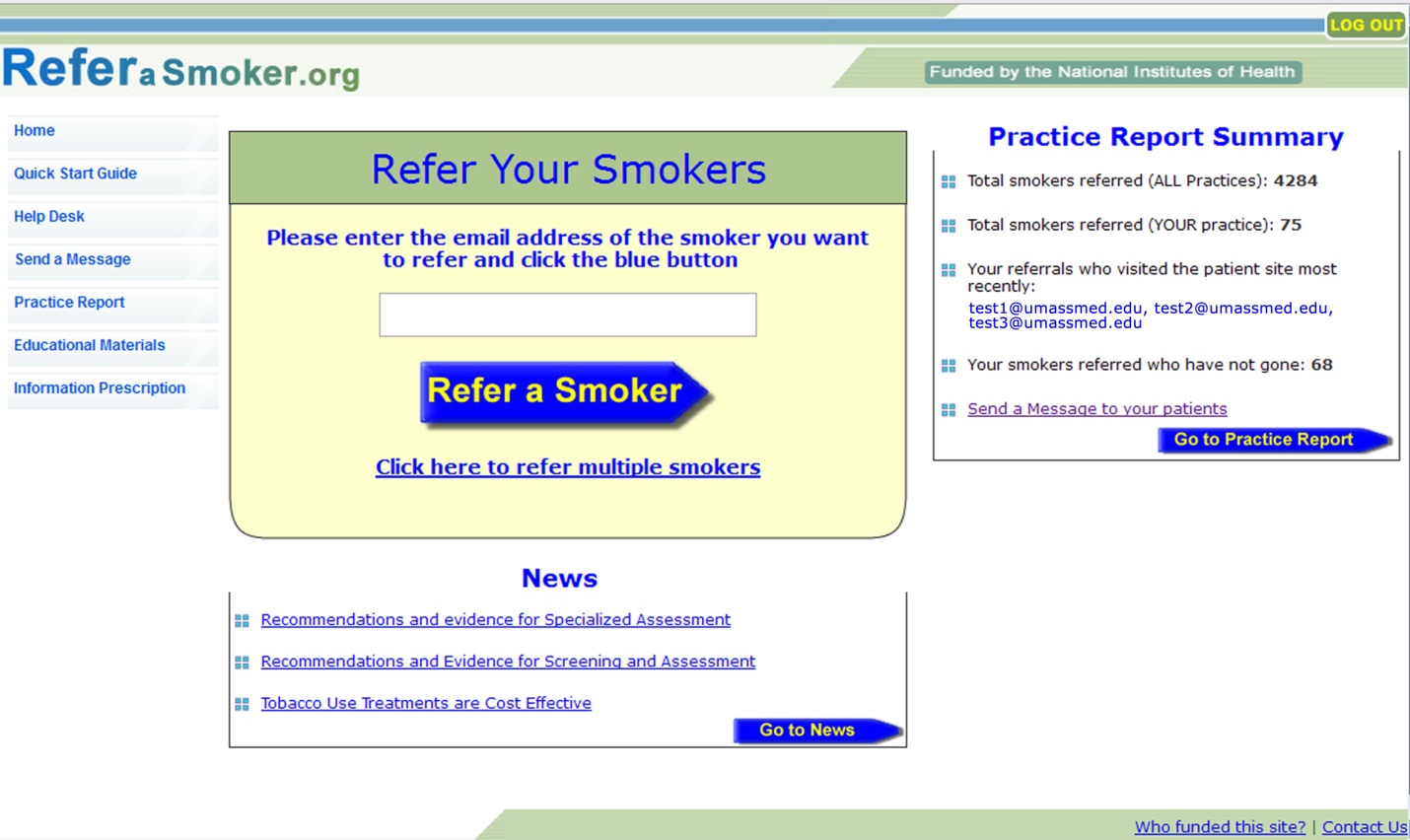


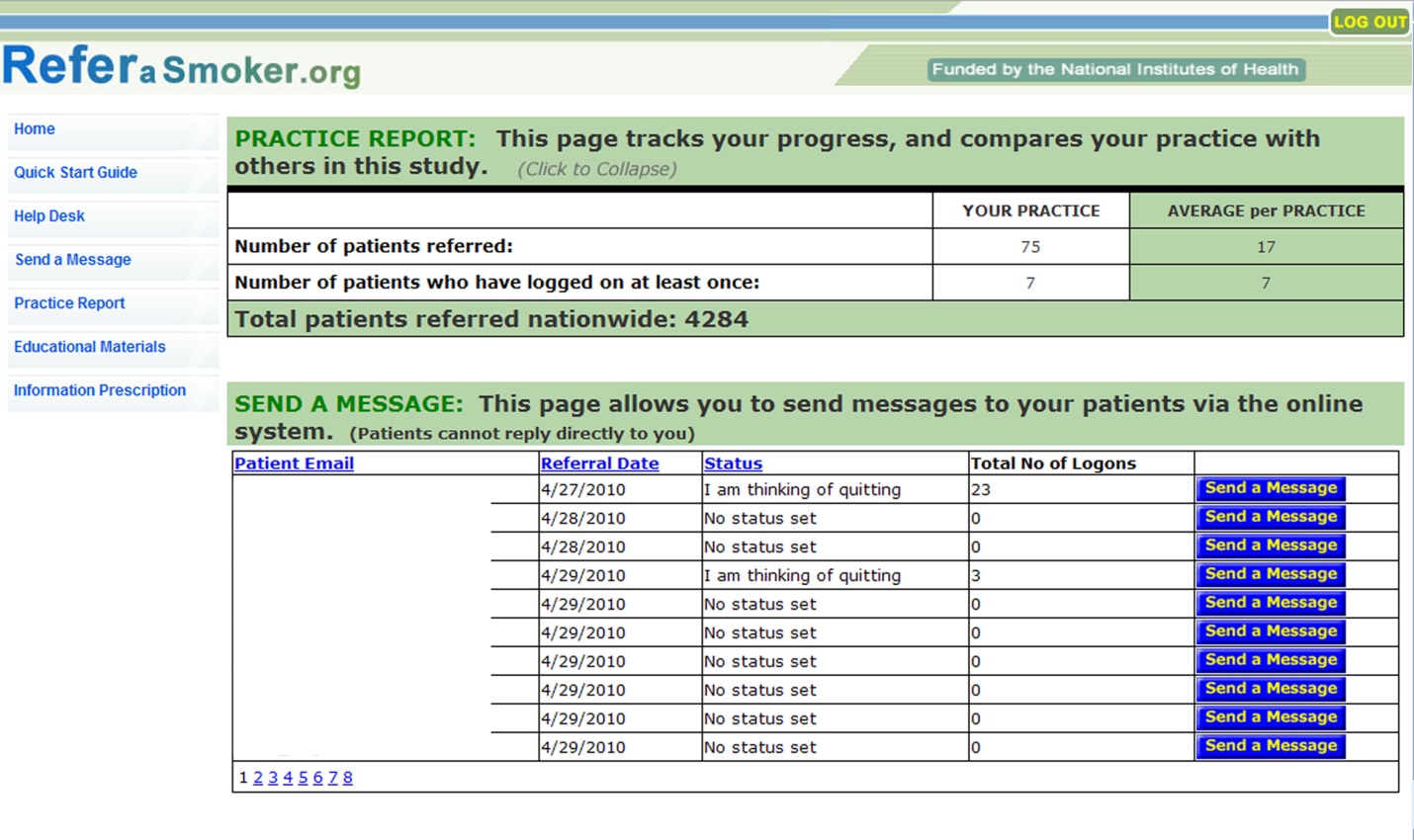

Supplement: Additional file 1: Trial 1. Comparing paper-referral and ePortal practice implementation, secure practice ePortal — .Screenshot of the homepage of ReferaSmoker.org, an interactive website to assist providers in helping their patients quit smoking. [file 13012_2015_336_MOESM1_ESM.docx]
