# Supplementary material for: Evaluating the QUIT-PRIMO clinical practice ePortal to increase smoker engagement with online cessation interventions: a national hybrid type 2 implementation study
Source: Implement Sci. 2015 Nov 2;10:154. doi: 10.1186/s13012-015-0336-8 (PMC4630887; doi:10.1186/s13012-015-0336-8)

**Appendix B: Trial 2: Clinical Effectiveness of the Web-Assisted Tobacco Intervention, Enhanced Patient Website**


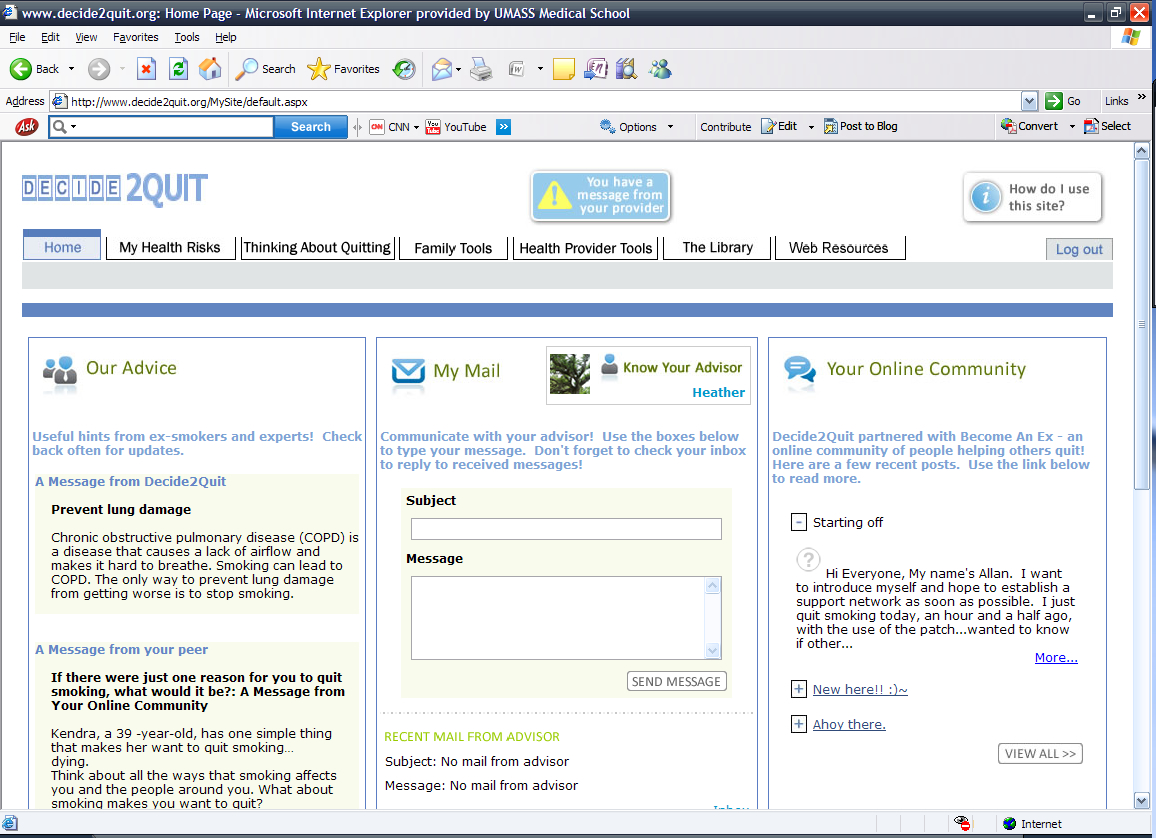

Supplement: Additional file 3: Trial 2. Clinical effectiveness of the web-assisted tobacco intervention, enhanced patient website. — Screenshot of the homepage of Decide2Quit.org, a multi-modal, evidence-based smoking cessation induction system available via the Internet. [file 13012_2015_336_MOESM3_ESM.docx]
