# Supplementary material for: Evaluating the QUIT-PRIMO clinical practice ePortal to increase smoker engagement with online cessation interventions: a national hybrid type 2 implementation study
Source: Implement Sci. 2015 Nov 2;10:154. doi: 10.1186/s13012-015-0336-8 (PMC4630887; doi:10.1186/s13012-015-0336-8)
Supplement: Additional file 5: Trial 2. — Clinical effectiveness of the web-assisted tobacco intervention, brief motivational messages. Examples of the brief motivational email messages that participants within the Messaging and Personalized groups received. [file 13012_2015_336_MOESM5_ESM.docx]

**Appendix C: Trial 2: Clinical Effectiveness of the Web-Assisted Tobacco Intervention, Brief Motivational Messages**

**Subject:** Breathing gets easier

**Message:** Everyone knows that smoking is bad for you. However, after you quit you may notice that you can breathe better and that you have more energy. Quitting also lowers your risk of getting cancer from smoking.

To learn more, please visit www.decide2quit.org.

**Subject:** Reasons to quit

**Message:** Need another reason to quit smoking? Quitting may help you feel better about yourself and will help keep your children healthier.

To learn more, please visit www.decide2quit.org.

**Subject:** Extend your lifespan

Did you know that smoking can cause heart disease, lung disease, all types of cancers, and can shorten your life by years?

To learn more, please visit www.decide2quit.org.

**Subject:** Man, I need a lift!

**Message:** Smoking depletes the skin’s natural glow and creates lines around the eyes, lips and cheeks. Quitting smoking can help reverse the harm that smoking has done to your skin.

To learn more, please visit www.decide2quit.org.

**Subject:** How to manage quitting

**Message:** Many people worry about managing without cigarettes. This is a normal concern and your provider is available to talk with you about this (or any other concerns you may have) about quitting.

To learn more, please visit www.decide2quit.org.

**Subject:** Quitting is worth the effort

**Message:** Your body may be dependent on the nicotine in tobacco, making it more difficult to quit smoking. Choosing to quit will present some challenging but worthwhile work. The benefits will be tremendous.

To learn more, please visit www.decide2quit.org.

**Subject:** What will you plan?

**Message:** You're ready to quit - fantastic! What do you plan to do to get ready for your quit date? Light exercise can be a great motivator for quitting and a great distracters after you quit to help you when you feel the urge to smoke. Check out the DecideQuit website for ideas from others who have quit smoking!

To learn more, please visit www.decide2quit.org.

**Subject:** Get up and get moving.

**Message:** You will be amazed at how much better you feel after you quit smoking. Make a list of physical activities you enjoy. Plan ahead to be more active after your quit date!

To learn more, please visit www.decide2quit.org.

**Subject:** You'll be surprised at how much energy.

**Message:** Has smoking ever made you stay away from physical activities? Many smokers do because they get short of breath or start coughing. When you quit smoking, you will probably notice it is easier to do more physical activities. If you are do not believe this, put it to the test yourself. Try some difficult physical activity now, and again after you quit. You will amaze yourself at your own abilities.

To learn more, please visit www.decide2quit.org.
