# Supplementary material for: Evaluating the QUIT-PRIMO clinical practice ePortal to increase smoker engagement with online cessation interventions: a national hybrid type 2 implementation study
Source: Implement Sci. 2015 Nov 2;10:154. doi: 10.1186/s13012-015-0336-8 (PMC4630887; doi:10.1186/s13012-015-0336-8)
Supplement: Additional file 6: Trial 2. — Clinical effectiveness of the web-assisted show tobacco intervention,characteristics of participants who completed a 6-month follow-up and did not complete a 6-month followup. Table of participant characteristics comparing those who did and did not complete the 6-month follow-up. [file 13012_2015_336_MOESM6_ESM.docx]

|  | Completed Follow-Up (n=466) | | Missing Follow-Up (n=434) | |
| --- | --- | --- | --- | --- |
|  | N | % | N | % |
| **Patient Sex** |  |  |  |  |
| Female | 288 | 62 | 282 | 65 |
| Male | 178 | 38 | 152 | 35 |
| **Patient Age** |  |  |  |  |
| 19-34 | 74 | 16 | 78 | 18 |
| 35-55 | 225 | 48 | 229 | 53 |
| 55-64 | 126 | 27 | 98 | 23 |
| 65+ | 41 | 9 | 29 | 7 |
| **Patient Race** |  |  |  |  |
| White | 399 | 86 | 370 | 85 |
| Black or African American | 51 | 11 | 36 | 8 |
| Others | 16 | 3 | 28 | 6 |
| **Patient Education** |  |  |  |  |
| Less than high school | 29 | 6 | 46 | 11 |
| High school graduate | 130 | 28 | 140 | 33 |
| Some college | 209 | 45 | 171 | 40 |
| College graduate or more | 94 | 20 | 73 | 17 |
| **Readiness to Quit** ‡ |  |  |  |  |
| Not thinking of quitting | 18 | 4 | 16 | 4 |
| Thinking of quitting | 370 | 82 | 329 | 83 |
| Set a quit date | 64 | 14 | 53 | 13 |
| **Allow smoking at home** |  |  |  |  |
| No | 215 | 50 | 245 | 53 |
| Yes | 219 | 50 | 221 | 47 |
| **Number of cigarettes per day** |  |  |  |  |
| 0-10 | 125 | 27 | 116 | 27 |
| 11-20 | 238 | 51 | 222 | 51 |
| >20 | 103 | 22 | 96 | 22 |
| **Visited other smoking cessation websites before** |  |  |  |  |
| No | 402 | 86 | 380 | 88 |
| Yes | 64 | 14 | 54 | 12 |
| **Quit attempt (1 day or more) in past 12 months** |  |  |  |  |
| No | 216 | 46 | 208 | 48 |
| Yes | 250 | 54 | 226 | 52 |

**Appendix D: Trial 2: Clinical Effectiveness of the Web-Assisted Tobacco Intervention, Characteristics of Participants who completed 6-month Follow-Up and did not complete 6-month Follow-Up**
